# [(2,3,5,6-η)-Bicyclo­[2.2.1]hepta-2,5-diene]dibromidopalladium(II)

**DOI:** 10.1107/S1600536809020583

**Published:** 2009-06-06

**Authors:** Nam-Ho Kim, Kwang Ha

**Affiliations:** a School of Applied Chemical Engineering, the Research Institute of Catalysis, Chonnam National University, Gwangju 500-757, Republic of Korea

## Abstract

In the title complex, [PdBr_2_(C_7_H_8_)], the Pd^II^ ion lies in a distorted square-planar environment defined by the two Br atoms and the mid-points of the two π-coordinated double bonds of bicyclo­[2.2.1]hepta-2,5-diene. The complex is disposed about a crystallographic mirror plane parallel to the *ac* plane passing through the Pd, Br atoms and the centre of the diene ligand.

## Related literature

For the preparation of [Pd*X*_2_(nbd)] (*X* = Cl or Br; nbd = (norbornadiene), see: Alexander *et al.* (1960 ▶). For the crystal structure of [PdCl_2_(nbd)], see: Baenziger *et al.* (1965 ▶). For the gas electron diffraction structure of norbornadiene, see: Yokozeki & Kuchitsu (1971 ▶).
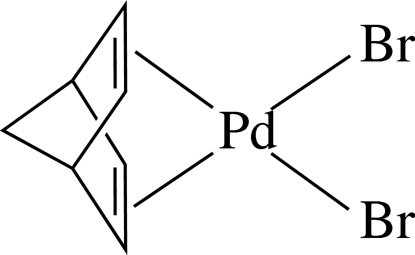


## Experimental

### 

#### Crystal data

[PdBr_2_(C_7_H_8_)]
*M* *_r_* = 358.35
Orthorhombic, 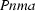

*a* = 12.758 (2) Å
*b* = 7.4313 (11) Å
*c* = 9.0138 (14) Å
*V* = 854.6 (2) Å^3^
*Z* = 4
Mo *K*α radiation
μ = 11.44 mm^−1^
*T* = 296 K
0.22 × 0.20 × 0.15 mm

#### Data collection

Bruker SMART 1000 CCD diffractometer
Absorption correction: multi-scan (*SADABS*; Bruker, 2000 ▶) *T*_min_ = 0.126, *T*_max_ = 0.180
5210 measured reflections
944 independent reflections
673 reflections with *I* > 2σ(*I*)
*R*_int_ = 0.042

#### Refinement

*R*[*F* ^2^ > 2σ(*F* ^2^)] = 0.026
*wR*(*F* ^2^) = 0.071
*S* = 0.99
944 reflections
52 parameters
H-atom parameters constrained
Δρ_max_ = 1.10 e Å^−3^
Δρ_min_ = −1.79 e Å^−3^

### 

Data collection: *SMART* (Bruker, 2000 ▶); cell refinement: *SAINT* (Bruker, 2000 ▶); data reduction: *SAINT*; program(s) used to solve structure: *SHELXS97* (Sheldrick, 2008 ▶); program(s) used to refine structure: *SHELXL97* (Sheldrick, 2008 ▶); molecular graphics: *PLATON* (Spek, 2009 ▶); software used to prepare material for publication: *SHELXL97*.

## Supplementary Material

Crystal structure: contains datablocks I. DOI: 10.1107/S1600536809020583/ez2172sup1.cif

Structure factors: contains datablocks I. DOI: 10.1107/S1600536809020583/ez2172Isup2.hkl

Additional supplementary materials:  crystallographic information; 3D view; checkCIF report

## Figures and Tables

**Fig. 1.**
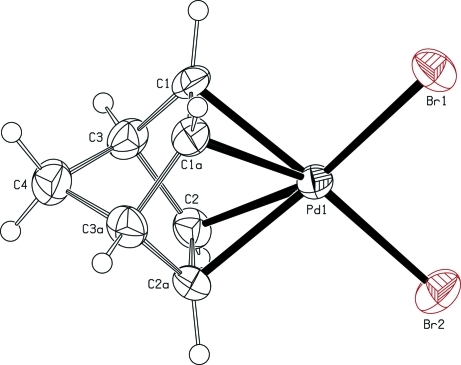
The structure of the title complex, with displacement ellipsoids drawn at the 40% probability level for non-H atoms [Symmetry code: (a) x, 1/2 - y, z].

**Fig. 2.**
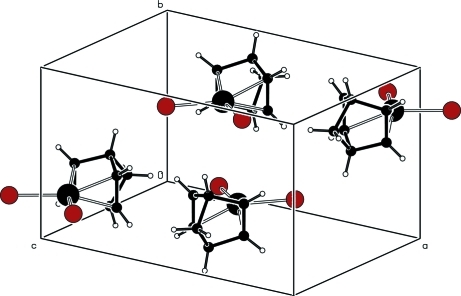
View of the unit-cell contents of the title complex.

## supplementary crystallographic information

### Comment

The title complex, [PdBr_2_(C_7_H_8_)], is isomorphous with the analogous Pd(II) complex [PdCl_2_(C_7_H_8_)] (Baenziger *et al.*, 1965). In the complex, the central Pd^II^ ion is essentially in a square-planar environment defined by the two Br atoms and the two midpoints (M1, M2) of the π-coordinated double bonds of the bicyclo[2.2.1]hepta-2,5-diene (norbornadiene; nbd) ligand [M1 and M2 denote the midpoints of the olefinic bonds C1—C1a and C2—C2a, respectively; symmetry code: (*a*) *x*, 1/2 - *y*, *z*] (Fig. 1). The complex is disposed about a crystallographic mirror plane parallel to the *ac* plane passing through the Pd atom, the Br atoms and the centre of the ligand with the special positions (*x*, 1/4, *z*) (Fig. 2). The pairs of Pd—Br and Pd—C bond lengths are almost equal (Pd—Br: 2.4258 (11) and 2.4294 (10) Å; Pd—C: 2.165 (5) and 2.170 (5) Å). The nbd ligand coordinates symmetrically to the Pd atom, and displays a slight increase in the double-bond distances (1.389 (10) and 1.388 (10) Å) compared with the non-coordinating double bonds of nbd in the gas phase (1.343 (3) Å; Yokozeki & Kuchitsu, 1971).

### Experimental

To a solution of (bicyclo[2.2.1]hepta-2,5-diene)dichloridopalladium(II) (0.200 g, 0.742 mmol) in EtOH (20 ml) was added NaBr (0.816 g, 7.931 mmol), and refluxed for 1 h. The formed precipitate was separated by filtration and washed with EtOH and water and dried under vacuum, to give an orange powder (0.053 g). Crystals suitable for X-ray analysis were obtained by slow evaporation from a CH_3_CN solution.

### Refinement

H atoms were positioned geometrically and allowed to ride on their respective parent atoms [C—H = 0.98 (CH) or 0.97 Å (CH_2_) and *U*_iso_(H) = 1.2*U*_eq_(C)].

### Crystal data

**Table:**

[PdBr_2_(C_7_H_8_)]	*F*(000) = 664
*M**_r_* = 358.35	*D*_x_ = 2.785 Mg m^−^^3^
Orthorhombic, *P**n**m**a*	Mo *K*α radiation, λ = 0.71073 Å
Hall symbol: -P 2ac 2n	Cell parameters from 2031 reflections
*a* = 12.758 (2) Å	θ = 2.7–27.9°
*b* = 7.4313 (11) Å	µ = 11.44 mm^−^^1^
*c* = 9.0138 (14) Å	*T* = 296 K
*V* = 854.6 (2) Å^3^	Block, orange
*Z* = 4	0.22 × 0.20 × 0.15 mm

### Data collection

**Table:**

Bruker SMART 1000 CCD diffractometer	944 independent reflections
Radiation source: fine-focus sealed tube	673 reflections with *I* > 2σ(*I*)
graphite	*R*_int_ = 0.042
φ and ω scans	θ_max_ = 26.4°, θ_min_ = 2.8°
Absorption correction: multi-scan (*SADABS*; Bruker, 2000)	*h* = −14→15
*T*_min_ = 0.126, *T*_max_ = 0.180	*k* = −8→9
5210 measured reflections	*l* = −11→6

### Refinement

**Table:**

Refinement on *F*^2^	Primary atom site location: structure-invariant direct methods
Least-squares matrix: full	Secondary atom site location: difference Fourier map
*R*[*F*^2^ > 2σ(*F*^2^)] = 0.026	Hydrogen site location: inferred from neighbouring sites
*wR*(*F*^2^) = 0.071	H-atom parameters constrained
*S* = 0.99	*w* = 1/[σ^2^(*F*_o_^2^) + (0.0331*P*)^2^] where *P* = (*F*_o_^2^ + 2*F*_c_^2^)/3
944 reflections	(Δ/σ)_max_ < 0.001
52 parameters	Δρ_max_ = 1.10 e Å^−^^3^
0 restraints	Δρ_min_ = −1.79 e Å^−^^3^

### Special details

**Table:**

Geometry. All e.s.d.'s (except the e.s.d. in the dihedral angle between two l.s. planes) are estimated using the full covariance matrix. The cell e.s.d.'s are taken into account individually in the estimation of e.s.d.'s in distances, angles and torsion angles; correlations between e.s.d.'s in cell parameters are only used when they are defined by crystal symmetry. An approximate (isotropic) treatment of cell e.s.d.'s is used for estimating e.s.d.'s involving l.s. planes.
Refinement. Refinement of *F*^2^ against ALL reflections. The weighted *R*-factor *wR* and goodness of fit *S* are based on *F*^2^, conventional *R*-factors *R* are based on *F*, with *F* set to zero for negative *F*^2^. The threshold expression of *F*^2^ > σ(*F*^2^) is used only for calculating *R*-factors(gt) *etc*. and is not relevant to the choice of reflections for refinement. *R*-factors based on *F*^2^ are statistically about twice as large as those based on *F*, and *R*- factors based on ALL data will be even larger.

### Fractional atomic coordinates and isotropic or equivalent isotropic displacement parameters (Å^2^)

**Table:**

	*x*	*y*	*z*	*U*_iso_*/*U*_eq_	
Pd1	0.06852 (4)	0.2500	0.92135 (7)	0.0312 (2)	
Br1	0.19165 (7)	0.2500	1.12643 (11)	0.0462 (3)	
Br2	−0.08564 (6)	0.2500	1.07957 (11)	0.0491 (3)	
C1	0.1765 (4)	0.1566 (7)	0.7528 (6)	0.0363 (14)	
H1	0.2388	0.0844	0.7752	0.044*	
C2	−0.0067 (4)	0.1566 (7)	0.7196 (6)	0.0380 (14)	
H2	−0.0709	0.0844	0.7192	0.046*	
C3	0.0934 (4)	0.0997 (8)	0.6402 (7)	0.0419 (15)	
H3	0.0967	−0.0241	0.6031	0.050*	
C4	0.1048 (6)	0.2500	0.5270 (11)	0.052 (2)	
H4A	0.1727	0.2500	0.4786	0.063*	
H4B	0.0492	0.2500	0.4536	0.063*	

### Atomic displacement parameters (Å^2^)

**Table:**

	*U*^11^	*U*^22^	*U*^33^	*U*^12^	*U*^13^	*U*^23^
Pd1	0.0263 (3)	0.0349 (4)	0.0325 (4)	0.000	0.0009 (3)	0.000
Br1	0.0491 (5)	0.0448 (5)	0.0448 (6)	0.000	−0.0147 (4)	0.000
Br2	0.0430 (5)	0.0488 (6)	0.0556 (7)	0.000	0.0181 (4)	0.000
C1	0.025 (2)	0.046 (3)	0.038 (4)	0.007 (2)	0.006 (2)	−0.005 (3)
C2	0.032 (3)	0.048 (3)	0.034 (3)	−0.006 (2)	−0.007 (3)	−0.002 (3)
C3	0.043 (3)	0.040 (3)	0.042 (4)	0.003 (3)	0.002 (3)	−0.013 (3)
C4	0.043 (5)	0.073 (7)	0.040 (6)	0.000	0.005 (4)	0.000

### Geometric parameters (Å, °)

**Table:**

Pd1—C1^i^	2.165 (5)	C2—C2^i^	1.388 (10)
Pd1—C1	2.165 (5)	C2—C3	1.523 (7)
Pd1—C2^i^	2.170 (5)	C2—H2	0.9800
Pd1—C2	2.170 (5)	C3—C4	1.520 (9)
Pd1—Br1	2.4258 (11)	C3—H3	0.9800
Pd1—Br2	2.4294 (10)	C4—C3^i^	1.520 (9)
C1—C1^i^	1.389 (10)	C4—H4A	0.9700
C1—C3	1.527 (7)	C4—H4B	0.9700
C1—H1	0.9800		
			
C1^i^—Pd1—C1	37.4 (3)	Pd1—C1—H1	123.2
C1^i^—Pd1—C2^i^	65.8 (2)	C2^i^—C2—C3	106.1 (3)
C1—Pd1—C2^i^	78.2 (2)	C2^i^—C2—Pd1	71.35 (14)
C1^i^—Pd1—C2	78.2 (2)	C3—C2—Pd1	96.4 (3)
C1—Pd1—C2	65.8 (2)	C2^i^—C2—H2	123.2
C2^i^—Pd1—C2	37.3 (3)	C3—C2—H2	123.2
C1^i^—Pd1—Br1	97.05 (14)	Pd1—C2—H2	123.2
C1—Pd1—Br1	97.05 (14)	C4—C3—C2	101.0 (5)
C2^i^—Pd1—Br1	157.65 (14)	C4—C3—C1	100.2 (5)
C2—Pd1—Br1	157.65 (14)	C2—C3—C1	101.1 (4)
C1^i^—Pd1—Br2	157.97 (13)	C4—C3—H3	117.2
C1—Pd1—Br2	157.97 (13)	C2—C3—H3	117.2
C2^i^—Pd1—Br2	97.71 (14)	C1—C3—H3	117.2
C2—Pd1—Br2	97.71 (14)	C3^i^—C4—C3	94.6 (7)
Br1—Pd1—Br2	94.41 (4)	C3^i^—C4—H4A	112.8
C1^i^—C1—C3	106.1 (3)	C3—C4—H4A	112.8
C1^i^—C1—Pd1	71.29 (13)	C3^i^—C4—H4B	112.8
C3—C1—Pd1	96.5 (3)	C3—C4—H4B	112.8
C1^i^—C1—H1	123.2	H4A—C4—H4B	110.3
C3—C1—H1	123.2		
			
C2^i^—Pd1—C1—C1^i^	65.45 (15)	C2^i^—Pd1—C2—C3	104.9 (3)
C2—Pd1—C1—C1^i^	102.59 (16)	Br1—Pd1—C2—C3	−40.3 (6)
Br2—Pd1—C1—C1^i^	146.8 (4)	Br2—Pd1—C2—C3	−162.5 (3)
C1^i^—Pd1—C1—C3	−104.8 (3)	C2^i^—C2—C3—C4	−33.3 (5)
C2^i^—Pd1—C1—C3	−39.3 (3)	Pd1—C2—C3—C4	−105.7 (4)
C2—Pd1—C1—C3	−2.2 (3)	C2^i^—C2—C3—C1	69.5 (4)
Br1—Pd1—C1—C3	162.8 (3)	Pd1—C2—C3—C1	−2.9 (4)
Br2—Pd1—C1—C3	42.0 (6)	C1^i^—C1—C3—C4	34.0 (4)
C1^i^—Pd1—C2—C2^i^	−65.40 (15)	Pd1—C1—C3—C4	106.4 (4)
C1—Pd1—C2—C2^i^	−102.69 (16)	C1^i^—C1—C3—C2	−69.5 (4)
Br1—Pd1—C2—C2^i^	−145.2 (4)	Pd1—C1—C3—C2	2.9 (4)
C1^i^—Pd1—C2—C3	39.5 (3)	C2—C3—C4—C3^i^	51.1 (6)
C1—Pd1—C2—C3	2.2 (3)	C1—C3—C4—C3^i^	−52.5 (6)

Symmetry codes: (i) *x*, −*y*+1/2, *z*.
